# Formulation and in vitro evaluation of self-nanoemulsifying liquisolid tablets of furosemide

**DOI:** 10.1038/s41598-020-79940-5

**Published:** 2021-01-14

**Authors:** Lena Dalal, Abdul Wahab Allaf, Hind El-Zein

**Affiliations:** 1 Faculty of Pharmacy, Damascus University, Damascus, Syria; 2 Faculty of Pharmacy, Arab International University, P.O. Box 16180, Ghabaghib, Daraa Syria

**Keywords:** Drug development, Nanomedicine

## Abstract

Self-nanoemulsifying drug delivery systems (SNEDDS) were used to enhance the dissolution rate of furosemide as a model for class IV drugs and the system was solidified into liquisolid tablets. SNEDDS of furosemide contained 10% Castor oil, 60% Cremophor EL, and 30% PEG 400. The mean droplets size was 17.9 ± 4.5 nm. The theoretical model was used to calculate the amounts of the carrier (Avicel PH101) and coating materials (Aerosil 200) to prepare liquisolid powder. Carrier/coating materials ratio of 5/1 was used and Ludipress was added to the solid system, thus tablets with hardness of 45 ± 2 N were obtained. Liquisolid tablets showed 2-folds increase in drug release as compared to the generic tablets after 60 min in HCl 0.1 N using USP apparatus-II. Furosemide loaded SNEDDS tablets have great prospects for further in vivo studies, and the theoretical model is useful for calculating the adequate amounts of adsorbents required to solidify these systems.

## Introduction

Class IV drugs of the Biopharmaceutical Classification System (BCS) present a great challenge in oral formulations due to their low solubility and permeability since solubility enhancement approaches alone may not be sufficient to enhance oral bioavailability of these drugs (e.g., amphotericin B, furosemide, acetazolamide, ritonavir, paclitaxel)^1^.

Self-emulsifying drug delivery systems (SEDDS) present a useful mean to enhance both solubility and permeability of both class II and IV drugs^2,3^. The drug is dissolved in a mixture of oil, surfactant and co-solvent which forms oil-in-water (o/w) micro- or nano-emulsion with the gastrointestinal aqueous fluids after oral administration under gentle agitation by the digestive system^4–7^. SEDDS promote the intestinal lymphatic transport of the drug and inhibit its enzymatic degradation and gut wall efflux, which increase the intracellular concentration of the drug, and reduce the variability in rate and extent of absorption^8–12^.

Liquisolid systems (LS) were also developed to enhance drugs solubility. The drug solution in non-volatile solvent is adsorbed on a carrier material, and then mixed with a coating material with high surface area to prepare a dry powder^13–15^.

Liquid SEDDS are usually filled in soft gelatine capsules which limited their widespread^16–18^. This led to the development of solid SEDDS by several methods into powder to be either filled in hard capsules or compressed into tablets^19,20^. However, the preparation of tablets is highly beneficial since hard capsules may only be filled up to about 400 mg due to the low bulk density of the carriers used (e.g. silicates)^21^.

Furosemide (FUR), a potent loop diuretic, is a class IV drug due to its low solubility in water (5–25 µg/ml) and low permeability^22,23^. The oral bioavailability of FUR is highly variable and the response to treatment is unpredictable with a large degree of differences within and between patients^24^.

The aim of this study was to optimize a hybrid liquisolid/self-emulsifying system of FUR as a model for class IV drugs and to prepare liquisolid tablets with acceptable properties.

## Materials and methods

### Materials

FUR was provided as a gift sample from UNIPHARMA pharmaceutical industries (AMRI, Aurangabad, India). Tween 80 was obtained from Riedel-De häen (Seelze-Hannover, Germany). Cremophor EL and Cremophor RH40 were a gift sample from Dar Al Dawa' pharmaceutical Co. Ltd (Amman, Jordan). Castor oil, Oleic acid, Glycerol, Propylene glycol (PG) and Polyethylene glycol 400 (PEG 400) were purchased from Panreac Co. Ltd (Barcelona, Spain). Sesame oil, Sunflower oil, and Soybean oil were purchased from the local market. Avicel PH101 was obtained from FMC Co. Ltd (Philadelphia, USA). Aerosil 200 was from Evonik industries (Rheinfelden, Germany). Ludipress was purchased from BASF (Ludwigshafen, Germany).

### Formulation of FUR loaded self-nanoemulsifying drug delivery system (FUR-SNEDDS)

#### Equilibrium solubility of FUR in excipients

In order to prepare FUR-SNEDDS, the required dose of FUR (20 mg) should dissolve in a small amount of the system. The solubility of FUR in various oils (Sesame oil, Castor oil, Sunflower oil, Soybean oil, Oleic acid), surfactants (Tween 80, Cremophor El, Cremophor RH40), and co-solvents (Glycerol, PG, PEG 400) was determined by shake flask method^25^. An excess amount of FUR was added to each capped tube containing 5 ml of each of the selected vehicles and was shaken at 25 °C for 48 h to reach equilibrium. Each tube was centrifuged at 6000 rpm (HERMLE Z200A, Germany) for 15 min. The supernatant was then filtered and diluted with methanol as necessary. The amount of soluble FUR was determined using UV-spectrophotometer (Scinco S-3100, Korea) at 274 nm, with methanol as a blank. All measurements were done in triplicates.

#### Pseudo-ternary phase diagram

The concentration of each component of the system that produced clear emulsions under mild stirring at room temperature (25 °C) was then determined using water titration method^26^. Three mixtures of surfactant/co-solvent (S/coS) were prepared in 3 ratios: A-mix (1/1), B-mix (2/1), and C-mix (1/2), then each mixture was mixed with the oil phase in 9 ratios (9:1, 8:2, 7:3, 6:4, 5:5, 4:6, 3:7, 2:8, 1:9). Water was added drop-wise under mild agitation by a magnetic stirrer for 15 min. The resulted mixtures were inspected visually for the formation of a clear/translucent emulsion that is associated with the formation of microemulsions^26^. The ratios that produced optically clear emulsions were plotted on a pseudo-ternary phase diagram using Tri-plot v1-2–4. No attempt was made to evaluate other phases when a white emulsion or gel-like phase was formed.

#### Preparation of FUR-SNEDDS

FUR-SNEDDS was prepared by dissolving FUR in sufficient amount of the self-emulsifying system (20 mg FUR/100 mg SNEDDS) and was sonicated for 30 min at 40 °C to facilitate solubilization. The droplets size and polydispersibility index (PdI) of the prepared system were measured using Zetasizer (Nano S, Malvern Instruments Ltd, UK) after diluting 1 ml with 100 ml of distilled water under gentle mixing by a magnetic stirrer.

### Formulation of LS powders

Hybrid LS compacts were prepared using the theoretical model^14^ to calculate the required amounts of the carrier and coating materials and produce flowable powders of FUR-SNEDDS as the liquid vehicle*.* Avicel PH101 and Aerosil 200 were used as carrier and coating materials, respectively.

The angle of slide (θ) was measured as described by Karmarkar et al.^27^, where uniform FUR-SNEDDS/powder admixtures that contained either Avicel PH101 or Aerosil 200 with increasing quantities of FUR-SNEDDS were prepared. The liquid/solid ratio that corresponded to an angle of slide of 33° was considered the flowable liquid-retention potential (Φ-value). Φ-value is the maximum amount of liquid that can be retained inside powder bulk (w/w) while maintaining acceptable flowability^27^.

Φ-value for SNEDDS of Avicel PH101 (ΦCa) and Aerosil 200 (ΦCo) were used to calculate liquid loading factor (Lf) with variant R-values (5, 10, 15, 20) using Eq. (1). The amount of carrier (Q) and coating (q) materials can be calculated by rearranging Eqs. (2) and (3) once the amount of liquid medication (W), and (R) values were determined^28^.

1 $${\text{Lf}} = \Phi {\text{Ca}} + \Phi {\text{Co}}\left( {1/{\text{R}}} \right)$$

2 $${\text{Lf}} = {\text{W}}/{\text{Q}}$$

3 $${\text{R}} = {\text{Q}}/{\text{q}}$$

Flowability of each LS compact with different R-ratios was evaluated using Carr’s compressibility index^29^.

### Reconstitution test

The ability of the hybrid LS compacts to re-emulsify spontaneously was tested by diluting an amount of LS powder that contained 1 ml of SNEDDS 100 times with water under mild agitation to form a clear emulsion and was then filtered to remove solids. The droplets size of the formed emulsion was measured using Zetasizer (Nano S, Malvern Instruments Ltd, UK).

### Preparation and characterization of hybrid LS tablets

**Figure 1:**
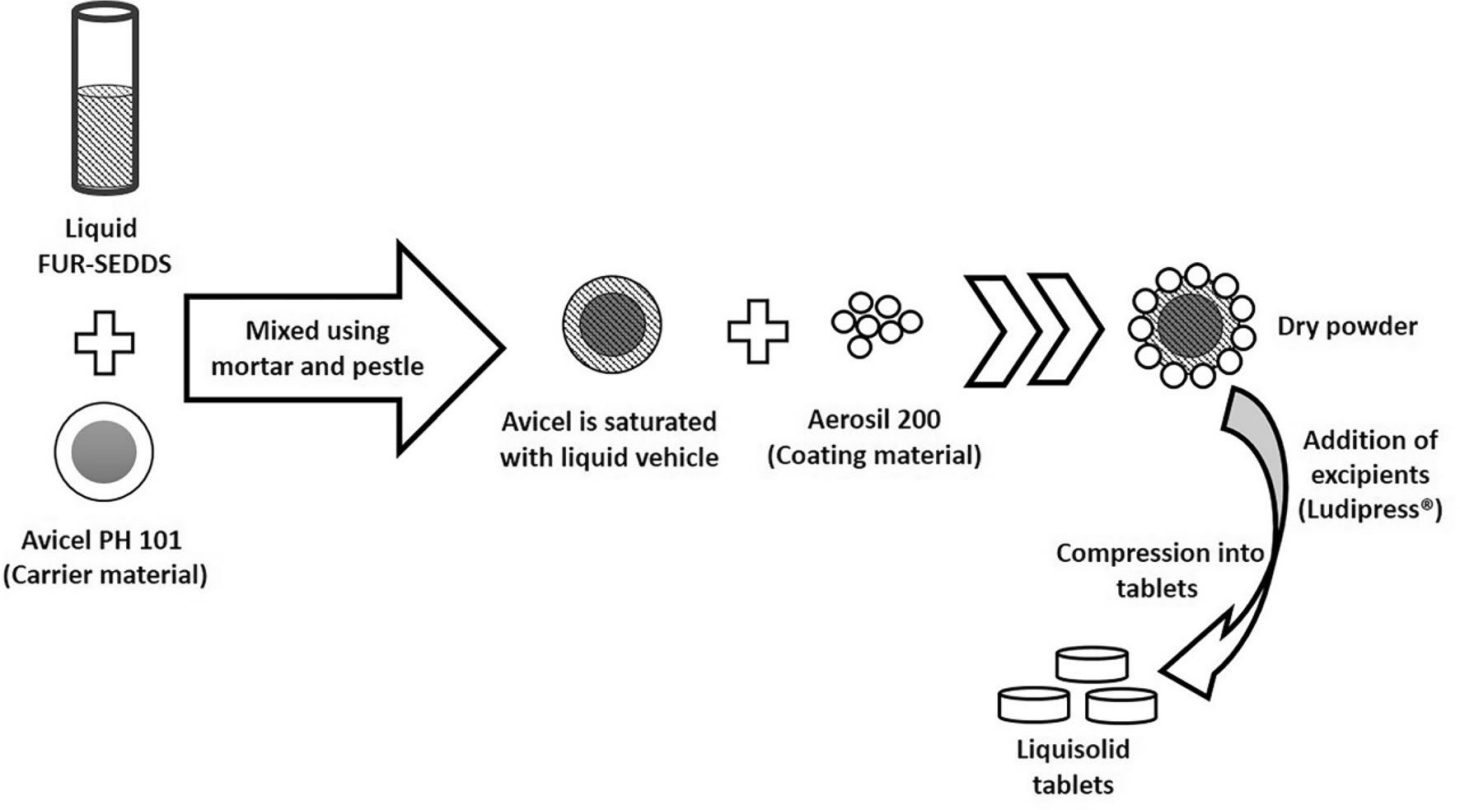
Schematic representation of the formulation of hybrid LS powder^30^.

The calculated amounts of Avicel PH101 and FUR-SNEDDS were blended using a mortar and pestle. The coating material (Aerosil 200) was then added to the mixture to obtain a dry LS powder. Ludipress was added to the LS powder as an adjuvant. A schematic representation of the preparation of the hybrid LS tablets (LST) is illustrated in Fig. 1^30^. Conventional directly compressed tablets (DCT) of FUR mixed with the powder components without the self-emulsifying system were prepared to evaluate the effect of self-emulsifying on drug release. The compositions of LST and DCT are shown in Table 1.

**Table 1:** The compositions of LST and DCT for 1 tablet.

Ingredients	LST	DCT
Furosemide	20 mg	20 mg
SNEDDS	100 mg	–
Avicel PH101	260 mg	260 mg
Aerosil 200	65 mg	65 mg
Ludipress	300 mg	300 mg

Single punch tablet machine (Erweka AR 402, Germany) with flat-faced punch was used for both tablets (LST and DCT). The hardness and friability of LST were evaluated using hardness (Erweka TBH 300, Germany) and friability testers (Erweka TAR 120, Germany).

### Differential Scanning Calorimeter (DSC)

The physical state of FUR in SNEDDS and LS powder was studied by DSC (Mettler Toledo TG50, Germany). Changes in melting enthalpy, glass transition temperature, and percentage of crystallinity due to any interactions with excipients were evaluated. Accurately weighed samples (2–10 mg) were placed in standard aluminium pans and the sample cell was purged with dry nitrogen at a flow rate of 100 ml/min. All samples were scanned from 25 to 400 °C at a heating rate of 10 °C/min*.*

### X-ray diffraction (XRD)

XRD patterns for FUR in LS powder and its pure components were recorded at room temperature (Stadi-P diffractometer, STOE, Germany). The samples were subjected to 40 kV voltage and 30 mA current conditions. The patterns were scanned over the angular range 3–90° (2θ) with 0.05° intervals and a counting time of 60 s per step.

### Scanning electron microscopy (SEM)

SEM imaging was utilized to examine the surface morphological characters of LS powder. The samples were mounted on a slab of metal with a double-sided adhesive carbon tape and were examined (VEGA-II, Tescan, Czech Republic).

### In-vitro dissolution studies

FUR release from LST in comparison to DCT and generic FUR tablets (GT) was determined by USP apparatus-II (DIS 8000, Copley, UK). The dissolution medium was 900 ml of either phosphate buffer pH 5.8 or HCl 0.1 N at 37 ± 0.5 °C, stirred at 100 rpm for 60 min. At the predetermined intervals (5, 10, 20, 30, 45, 60 min), 5 ml aliquots were withdrawn, filtered through a 0.45 µm membrane filter and assayed spectrophotometrically (Scinco S-3100, Korea) at 274 nm. Measurements were done in triplicates. Two-tailed student's t-test was performed to evaluate the significant differences in FUR release from LST and DCT or GT. The difference was considered statistically significant at *p-value* < 0.05.

## Results and discussion

**Figure 2:**
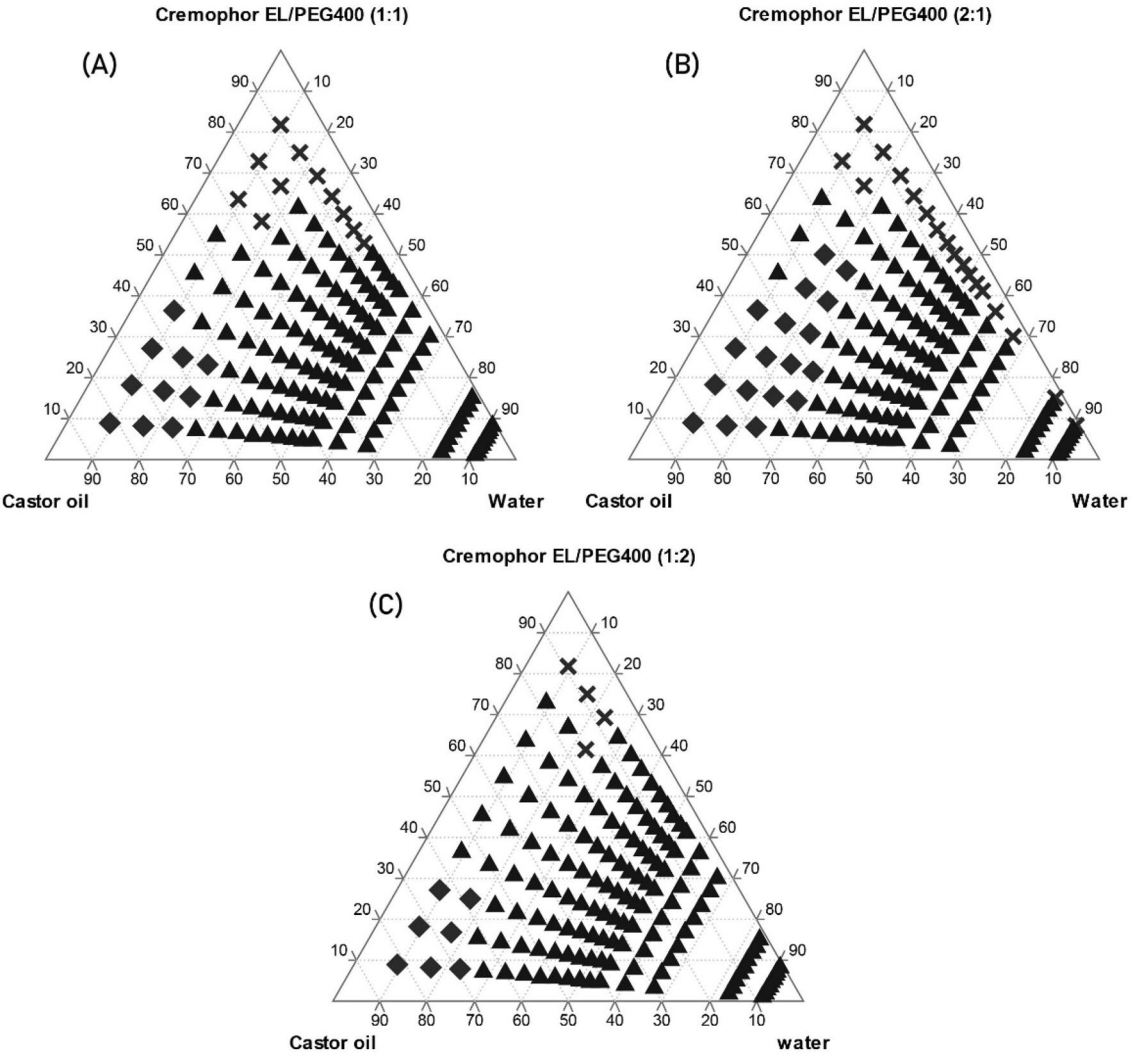
Pseudo-ternary phase diagrams of Cremophor El /PEG400:Castor oil:water with different Cremophor El/PEG400 ratios: (A)1:1, (B)2:1 and (C)1:2. (**X**) Represents the concentrations at which a clear/translucent emulsion was formed. (diamond) Gel-like phase. (triangle) White emulsion.

The solubility of FUR in each phase is displayed in Table 2. The highest solubility of FUR was in Castor oil (1.23 ± 0.045 mg/g) and Oleic acid (1.1 ± 0.057 mg/g) as oily phase, and in Cremophor EL (47.62 ± 2.227 mg/g) and PEG 400 (158.48 ± 6.379 mg/g) as surfactant and co-solvent, respectively. Therefore, the pseudo-ternary phase diagrams consisted of Cremophor El/PEG400 (S/coS) in different ratios: A-mix (1:1), B-mix (2:1), and C-mix (1:2) with Castor oil. The concentrations at which a clear/translucent emulsion was formed are represented as (**X**) in the diagrams in Fig. 2. Oleic acid was excluded from the results since its mixture with (S/coS) did not form a clear emulsion at any ratios.

**Table 2:** The solubility of FUR in various oils, surfactants, and co-solvents (mean ± SD, n = 3).

Vehicle	Solubility (mg/1 g) ± SD
Soybean oil	0.06 ± 0.00
Sunflower oil	0.06 ± 0.00
Castor oil	1.23 ± 0.04
Oleic acid	1.10 ± 0.06
Sesame oil	0.23 ± 0.02
Tween 80	31.4 ± 0.93
Cremophor RH40	30.71 ± 1.17
Cremophor El	47.62 ± 2.23
Glycerol	0.87 ± 0.01
PG	13.17 ± 0.55
PEG-400	158.48 ± 6.38

The diagrams show that the system consisting of 90% B-mix with 10% Castor oil formed a clear emulsion even after the addition of water up to 90% (Fig. 2B). On the other hand, for A-mix and C-mix the emulsion turned white after water addition up to 50% and 40%, respectively (Fig. 2A,C). This may be due to the higher concentration of Cremophor El (60%) as surfactant in B-mix, which formed a nano-emulsion upon water dilution with mean droplet size of (17.9 ± 4.5 nm), and PdI was 0.064. PdI below 0.3 indicates a good droplets size distribution. The droplets size was reported to have an impact on drug absorption since the smaller is the droplets size the larger is the interfacial surface for absorption^31^.

The solubility of FUR in the SNEDDS was (210.8 ± 26.6 mg/ml, n = 3), which is mainly attributed to the use of PEG 400 in the system as co-solvent.

### Preparation of FUR-SNEDDS LS powders

**Figure 3:**
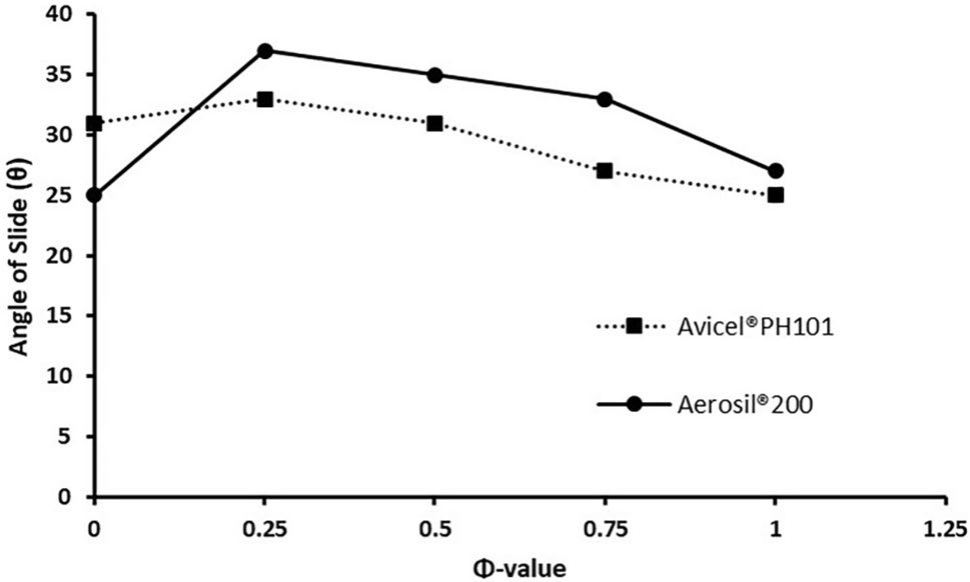
Angle of slide for admixtures of Avicel PH101 and Aerosil 200 SNEDDS as the liquid vehicle in different ratios.

The measured angle of slide of each admixture was plotted against Φ-value of either Avicel PH101 or Aerosil 200 with SNEDDS as the liquid vehicle in Fig. 3. Φ-values that corresponded to the angle of slide of 33° were 0.25 and 0.75 for Avicel PH101 (ΦCa) and Aerosil 200 (ΦCo), respectively. These values were used to calculate Lf using the Eqs. (1–3), where the amount of liquid FUR-SNEDDS (W) was considered (120 mg) in a dosage unit, as each unit contained 20 mg FUR dissolved in 100 mg SNEDDS. Several admixtures with variant R-values (5, 10, 15, 20) were prepared and their flowability and compressibility were evaluated using Carr's compressibility index as demonstrated in Table 3. LS-1 powder with the highest Aerosil 200 amount displayed the best flowability with Carr's index of 15.38% (Table 3). Increasing the amount of Aerosil 200 enhanced the flow properties of LS powder. This may be due the large surface area of Aerosil 200 that covers the surface of carrier particles and works as a lubricant and enhance powder flowability.

**Table 3:** The compositions and compressibility indexes of LS compacts.

LS compact	R value	Lf	Avicel PH101 (mg)	Aerosil 200 (mg)	CI* (%)
LS-1	5	0.4	300	60	15.38
LS-2	10	0.325	369.23	36.92	27.27
LS-3	15	0.3	400	26.67	27.91
LS-4	20	0.2875	417.39	20.87	26.67

*CI Carr's compressibility index.

### Reconstitution test

The mean droplets size of the reconstituted hybrid LS system was (19.82 ± 2.71 nm). The hybrid LS system maintained its ability to form a nanoemulsion after adsorption onto the solid carrier. PdI increased to (0.137) compared to the liquid system after water dilution, but still indicates a good uniformity in droplets size (PdI < 0.3)^31^.

### Characterization of LST

The adsorbents used to solidify SNEDDS have large surface area and consequently have low bulk density which presents a challenge upon filling the required quantities in hard gelatine capsules. However, tablets preparation holds some challenges due to the high compressibility index of silicates and squeezing out of liquids under compression that would lead to fragile tablets; especially at high liquid loads^17^. Decreasing drug load by increasing the powder quantity would significantly increase tablets size and weight to more than 1 g which makes it harder to swallow.

In the present study, Avicel PH101 and Aerosil 200 were employed as carrier and coating materials, respectively. Avicel PH101 is a commonly used carrier in liquisolid tablets formulation due to its good compactability^32^. Microcrystalline cellulose (Avicel) exhibits plastic deformation under compression, its microcrystalline nature and the hydrogen bonds between cellulose molecules gives tablets cohesion and strength^15^. On the other hand, very fine silica derivatives powders like Aerosil 200 with high adsorption properties are employed as coating materials in liquisolid compacts. The coating material (e.g. Aerosil 200) covers the wet carrier particles saturated with liquid to produce dry flowable powder^33^. Increasing the amount of Aerosil 200 in LS-1 increased Lf, which lowered the amount of powders needed to solidify the emulsifying system. To overcome the compressibility issues of Aerosil 200, an adjuvant was added. Kollidon K30 and Methocel K4M were respectively added to the LS powder in 1:1 ratio with no significant changes in powder compressibility (data not shown).

Ludipress (a mixture of Lactose monohydrate, Povidone, and Crospovidone) was added in increased ratios and tested. When Ludipress was added in 1:1 ratio to LS powder (see Table 2), tablets hardness and friability were 45 ± 2 N and 0.53%, respectively. The mean weight of LST unit was (745.62 ± 11.67 mg, n = 10) and none of the tablets showed a percentage deviation from the mean weight more than ± 5%. The prepared LST fulfilled the pharmacopeial standards of USP-38. Drug content was (99.81%) and within the accepted limits of 95.0–105.0%.

### DSC

**Figure 4:**
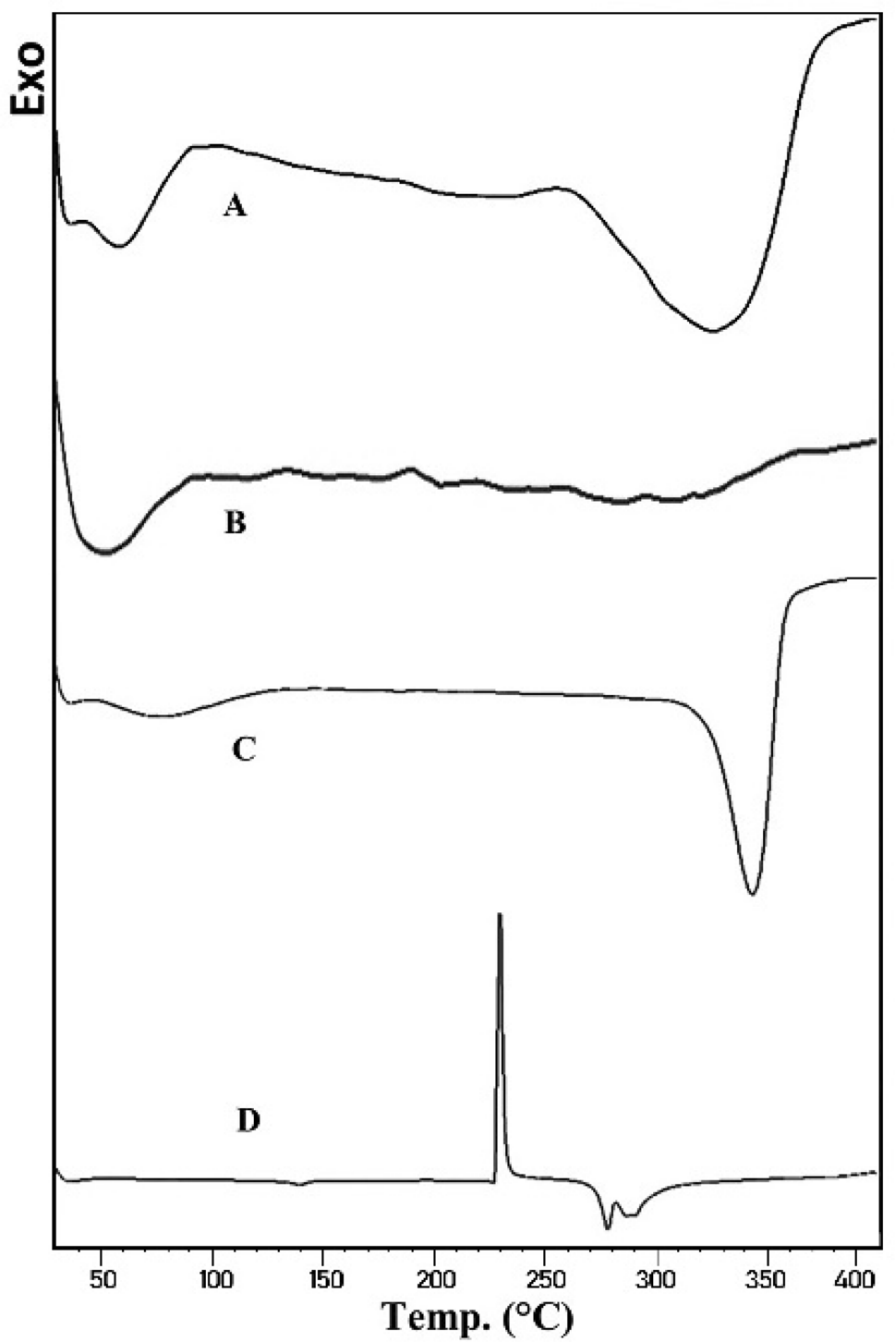
DSC thermograms of: (A) LS powder, (B) Aerosil 200, (C) Avicel PH101, (D) FUR.

The thermogram of FUR in Fig. 4D showed a big sharp exothermal peak at 226.94 °C due to its decomposition at its melting temperature^34–37^. Aerosil 200 is an amorphous material, the thermogram only showed a broad shallow feature due to the evaporation of adsorbed water (Fig. 4B). Similar observation was noticed for Avicel PH101 (Fig. 4C) at 50° to 120 °C before its melting at about 285 °C. The thermogram of LS powder (Fig. 4A) lacked the distinguishing exothermal peak of FUR with broadened endothermic peak of Avicel PH101. The disappearance of drug features in LS formulation indicates the formation of an amorphous solid solution as the drug is molecularly dispersed in LS system^38,39^.

### XRD

**Figure 5:**
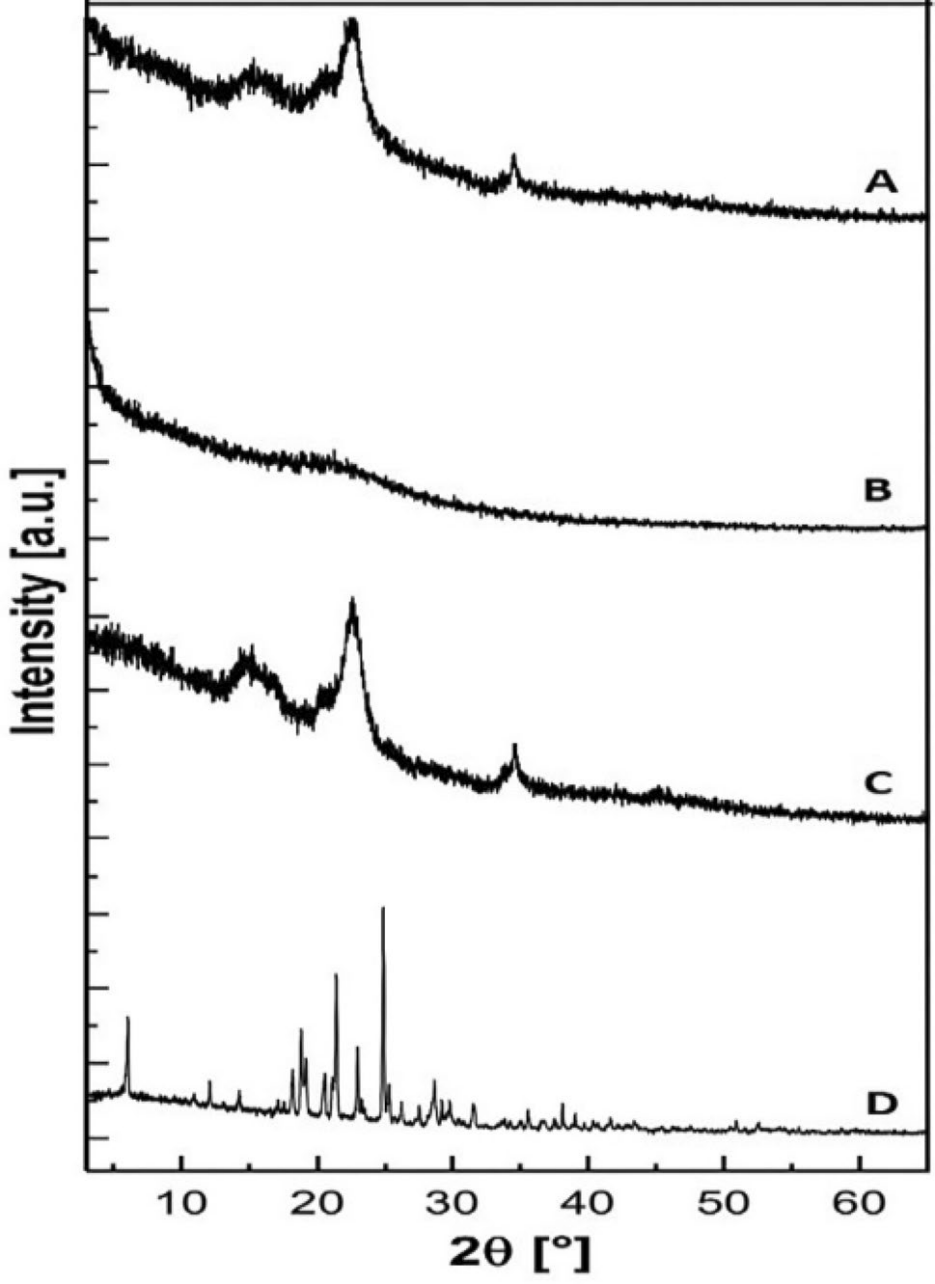
XRD patterns of (A) LS powder, (B) Aerosil 200, (C) Avicel PH101, (D) FUR.

XRD patterns demonstrate the crystalline nature of FUR and the solid components of LS powder (Fig. 5). The diffraction pattern of FUR (Fig. 5D) exhibited its crystalline nature with sharp and well-defined peaks. Avicel PH101 showed a typical microcrystalline pattern with broad peaks due to the small crystallites (Fig. 5C). Aerosil 200 is amorphous, thus the pattern has no defined features that indicate a crystalline module (Fig. 5B). The diffraction pattern of LS powder in (Fig. 5A) showed the broad peaks of Avicel PH101 with a small decline in its intensity. The disappearance of FUR features could be due to its conversion from crystalline to amorphous state. These results are in agreement with the previously mentioned DSC results and implicate that FUR is solubilized in the emulsifying system^40^.

### SEM

**Figure 6:**
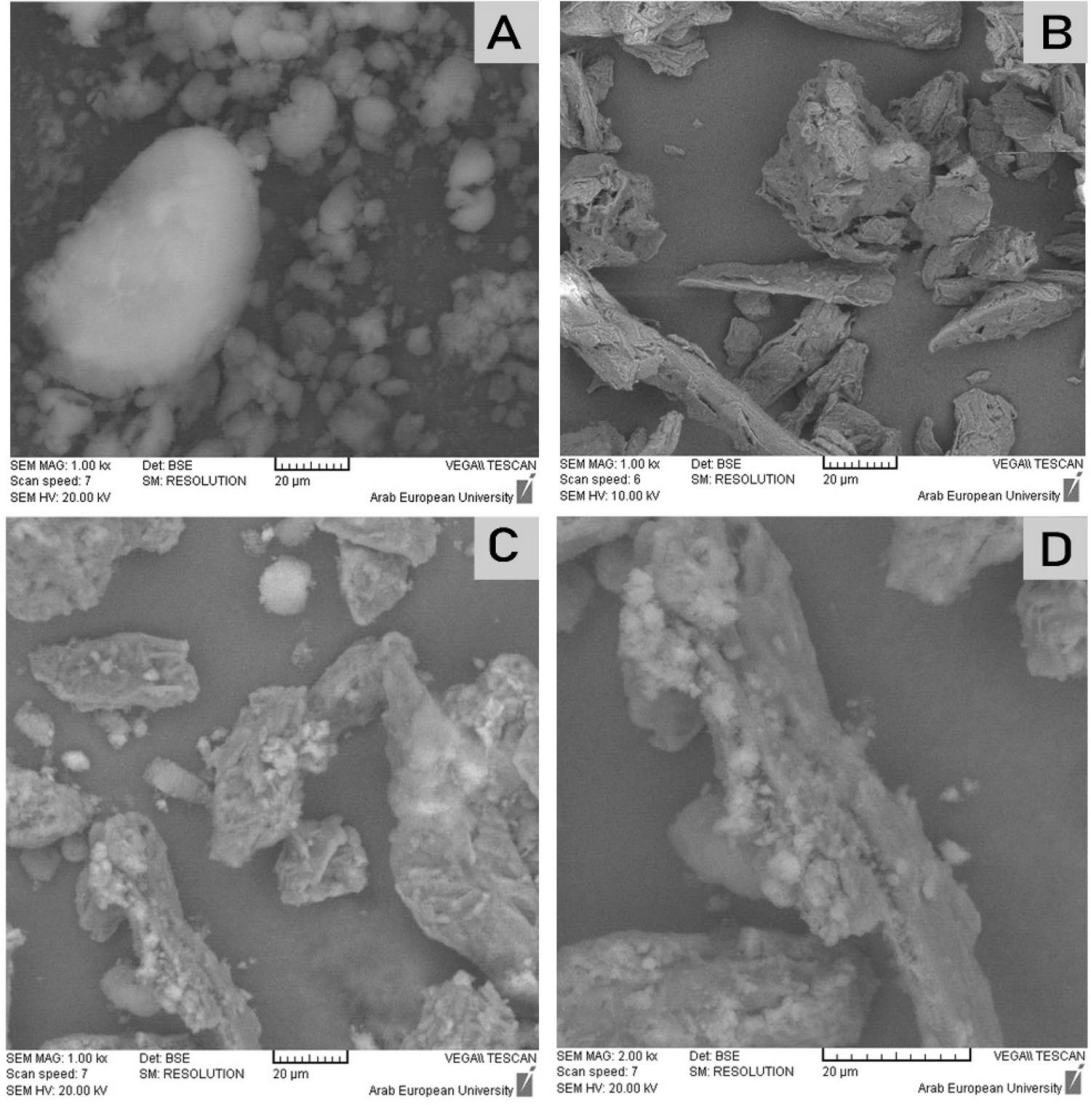
SEM images of (A) Aerosil 200, 1000x. (B) Avicel PH101, 1000x. (C) LS powder, 1000x, (D) LS powder, 2000x.

SEM images in Fig. 6C,D showed dry LS powder. This implicates; according to the liquisolid theory^30^, that the liquid system is adsorbed onto Avicel PH101 (the carrier material shown in Fig. 6B) and Aerosil 200 (the coating material shown in Fig. 6A) is adsorbed on Avicel particles that appears as a coating layer to form dry surface (see Fig. 1). This would interpret the compressibility index of LS-1 powder as Aerosil 200 acted as a lubricant and enhanced the powder's flowability.

### In-vitro dissolution studies

**Figure 7:**
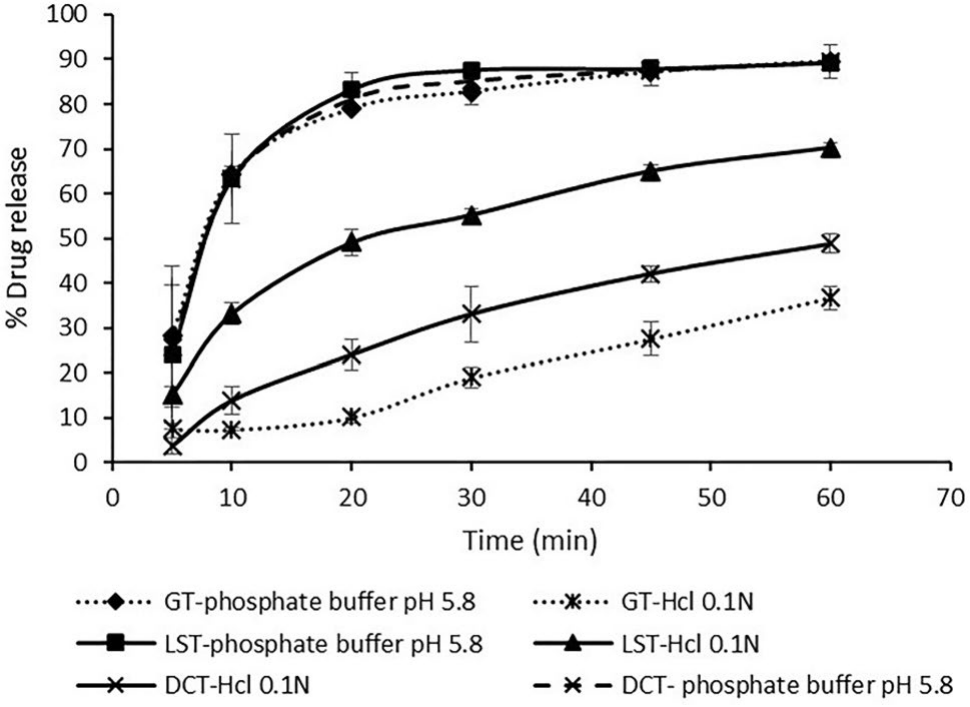
Percentage drug release of FUR from various tablets against time (min) in different mediums. Data are expressed as mean ± SD (n = 3).

The percentages of FUR released from LST, GT and DCT in different mediums are illustrated in Fig. 7. Both LST and GT released about 89% of FUR after 60 min with similar release profiles (*P* > 0.05, n = 3) in phosphate buffer pH 5.8. This may be attributed to the solubility of FUR in the dissolution medium due to its weak acidic properties (pKa ~ 3.9)^24^. This would lead to rapid absorption of FUR in the stomach, and the absorption is slowed down along the rising pH in the gastrointestinal tract from 3 to 5^41^.

Therefore, FUR release was studied in HCl 0.1 N as a dissolution medium. The release profile of LST was significantly different (*P* < 0.05, n = 3) from both GT and DCT release profiles. LST released 70.3% of FUR after 60 min, and 50% of FUR was released in 20 min. GT and DCT released about 36.7% and 48.8% of FUR after 60 min, respectively.

The self-emulsifying technique enhanced the solubility of FUR in the acidic medium up to 2-folds. The formation of nano-sized droplets upon mixing with the dissolution medium provided a large interfacial surface area, and presented FUR in a dissolved form^9^. Moreover, FUR is in the more soluble amorphous state in the LS compacts, which increased its solubility in the dissolution medium.

## Conclusion

LST demonstrated 2-folds increment in FUR release in the acidic medium (HCl 0.1 N), thus, have great potentials for further in vivo studies because of its higher dissolution in the medium where FUR is mostly absorbed. LS theoretical model is useful in calculating the adequate amounts of adsorbents required to solidify these systems, and the addition of Ludipress in tablets' formulation helped to overcome the poor compressibility characters of Aerosil 200.
